# Ground-Based Mobile Measurements to Track Urban Methane Emissions from Natural Gas in 12 Cities across Eight Countries

**DOI:** 10.1021/acs.est.3c03160

**Published:** 2024-01-25

**Authors:** F. Vogel, S. Ars, D. Wunch, J. Lavoie, L. Gillespie, H. Maazallahi, T. Röckmann, J. Nęcki, J. Bartyzel, P. Jagoda, D. Lowry, J. France, J. Fernandez, S. Bakkaloglu, R. Fisher, M. Lanoiselle, H. Chen, M. Oudshoorn, C. Yver-Kwok, S. Defratyka, J. A. Morgui, C. Estruch, R. Curcoll, C. Grossi, J. Chen, F. Dietrich, A. Forstmaier, H. A. C. Denier van der Gon, S. N. C. Dellaert, J. Salo, M. Corbu, S. S. Iancu, A. S. Tudor, A. I. Scarlat, A. Calcan

**Affiliations:** † Climate Research Division, Environment and Climate Change Canada, Toronto M3H 5T4, Canada; ‡ Department of Physics, University of Toronto, Toronto M5S 1A7, Canada; § Institute for Marine and Atmospheric Research Utrecht, Utrecht University, Utrecht 3584 CC, The Netherlands; ∥ AGH, University of Kraków, Kraków 30-059, Poland; ⊥ Department of Earth Sciences, Royal Holloway University of London, Egham, Surrey TW20 0EX, U.K.; # Centre for Isotope Research, Energy and Sustainability Research Institute, University of Groningen, Groningen 9747 AG, Netherlands; ¶ LSCE, CEA-CNRS-UVSQ, University Paris-Saclay, Gif-sur-Yvette 91191, France; ∇ ICTA, Autonomous University of Barcelona, Barcelona 08193, Spain; ○ Eurecat, Centre Tecnològic de Catalunya, Barcelona 08290, Spain; ⧫ INTE, Universitat Politècnica de Catalunya, Barcelona 08028, Spain; †† Environmental Sensing and Modelling, Technical University of Munich, Munich 80333, Germany; ‡‡ Netherlands Organisation for Applied Scientific Research—TNO, Utrecht 3584CB, The Netherlands; §§ Geography and GIS, University of Northern Colorado, Greeley, Colorado 80639, United States; ∥∥ Faculty of Physics, University of Bucharest, Bucharest 050663, Romania; ⊥⊥ INCAS, National Institute for Aerospace Research “Elie Carafoli”, Bucharest 061126, Romania

**Keywords:** methane, natural gas, mobile surveys, cities, greenhouse gas mitigation

## Abstract

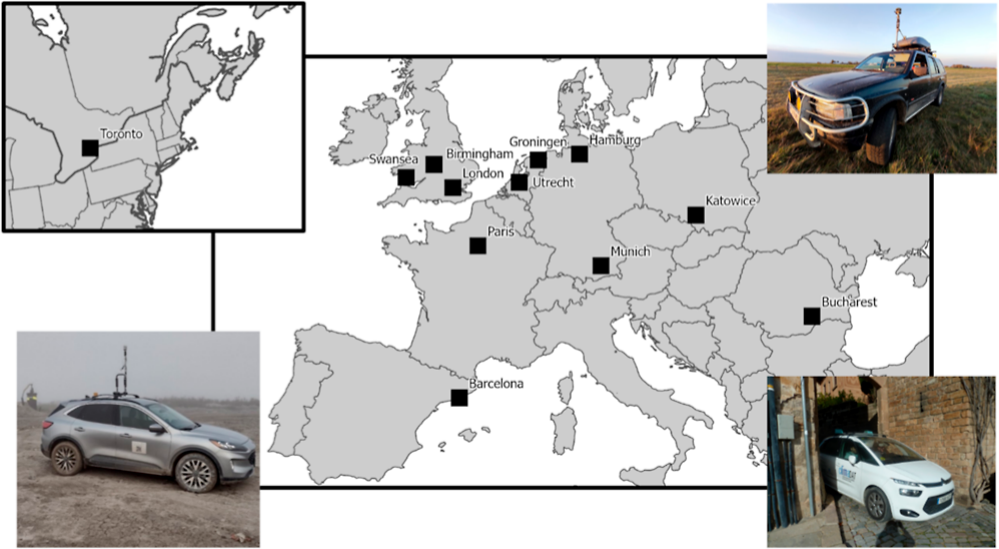

To mitigate methane emission from urban natural gas distribution systems, it is crucial to understand local leak rates and occurrence rates. To explore urban methane emissions in cities outside the U.S., where significant emissions were found previously, mobile measurements were performed in 12 cities across eight countries. The surveyed cities range from medium size, like Groningen, NL, to large size, like Toronto, CA, and London, UK. Furthermore, this survey spanned across European regions from Barcelona, ES, to Bucharest, RO. The joint analysis of all data allows us to focus on general emission behavior for cities with different infrastructure and environmental conditions. We find that all cities have a spectrum of small, medium, and large methane sources in their domain. The emission rates found follow a heavy-tailed distribution, and the top 10% of emitters account for 60–80% of total emissions, which implies that strategic repair planning could help reduce emissions quickly. Furthermore, we compare our findings with inventory estimates for urban natural gas-related methane emissions from this sector in Europe. While cities with larger reported emissions were found to generally also have larger observed emissions, we find clear discrepancies between observation-based and inventory-based emission estimates for our 12 cities.

## Abstract 2

We found significant methane leaks from the natural gas infrastructure in 12 cities and that the biggest 10% of sources account for 60–80% of the emissions in each city, which offers potential for cheap and rapid emission reductions for local stakeholders and companies.

## 1 Introduction

Despite global efforts to limit global warming, atmospheric greenhouse gas (GHG) concentrations continue to increase due to anthropogenic activities. Cities and metropolitan regions are key areas where current and future mitigation efforts need to be implemented to help reduce GHG emissions to achieve the Paris Agreement goal of limiting climate change to 1.5 °C, as they are responsible for 67–72% of global GHG emissions.^1^ In recent years, it has become apparent that mitigation of methane plays a crucial role, due to its large global warming potential (28–36 for the 100 year period) and short atmospheric lifetime (∼9a).^2,3^ Methane mitigation moved further into the spotlight during the COP26 in Glasgow as more than 100 countries joined the global methane pledge, which aims to reduce global methane emissions by 30% in 2030 relative to 2020 levels. One can also expect mitigation efforts for fossil fuel-related methane to be met with less hesitation compared to mitigation of fossil fuel carbon dioxide emissions as methane mitigation can even be economically profitable (e.g., refs (4) and (5)).

The effectiveness of local mitigation measures depends on understanding where major methane sources are located. For Organization for Economic Cooperation and Development (OECD) countries, the location of major facilities, such as landfills, wastewater treatment plants, or power plants, is often publicly available through national greenhouse gas reporting protocols/systems, which frequently include emission rate estimates (for example, https://www.epa.gov/ghgreporting). A recent report by the Clean Air Task Force highlighted that methane emissions from Europe’s oil and gas network are widespread^6^ but did not provide a quantification that can be compared to inventories. Aside from emissions from large oil and gas facilities, emissions in the distribution grid and at the consumer level are also important but pose different challenges to quantifying emissions, e.g., due to the densely populated city environments, vast lengths of underground pipework, and huge number of joints, as well as frequent physical obstructions which limit direct access.

Leaks and vents in urban natural gas distribution systems can occur over the whole city domain, thus complicating estimating citywide emissions. Previous surveys in urban areas in the United States revealed that methane emissions from natural gas infrastructure are responsible for a surprisingly large fraction of overall local emissions.^7^ A key finding by von Fischer et al. was that leak rates in cities with a large fraction of older, corrosion-prone pipeline materials were up to 25 times higher than those in cities with more modern infrastructure (e.g., Indianapolis, IN, US). The studies from high-emissions cities, like Boston, MA, US, clearly indicate that there is a significant mitigation potential regarding methane at the urban scale through infrastructure upgrades. In 2019, Weller et al.^8^ further investigated the underlying parameters to explain emission rates across US cities. A key finding was that emissions increase with pipeline age. Within the same age class, emissions were strongly determined by materials, with lowest emissions from plastic and coated steel pipelines and higher emissions from bare iron or cast-iron infrastructure. However, it is not apparent if those findings can be extended to other global regions. Recently studies from Europe have become available for, e.g., Hamburg, DE, Utrecht, NL,^9^ Paris, FR,^10,11^ and Bucharest, RO,^12^ most of which were included in this synthesis analysis. Unfortunately, only a couple of studies have been published for regions outside the US or EU (e.g., ref (13)), so a larger sample of city data is needed for a robust understanding of urban methane emissions globally. Another limitation in deriving comparable results is that no globally accepted measurement and data analysis methodology exists to date. Initial work in US cities often used bespoke methodologies. In recent years, different international organizations such as the United Nations Environmental Program as well as the World Meteorological Organizations Integrated Global Greenhouse Gas Information System have started to create science-based recommendations for urban methane surveys (e.g., https://library.wmo.int/viewer/58055/) and private sector actors have published their protocols (https://veritas.gti.energy/protocols). However, to date, no fully developed international standard exists. The most used approach remains the open-source methodology developed by Weller et al.^8^

This study synthesizes mobile measurements collected between 2018 and 2020 in 12 cities in Europe and Canada to investigate whether significant methane emissions from urban natural gas infrastructure are common, whether overall emissions are similar to reported inventories, and whether rare but strong emitters dominate the emission landscape. The cities included here span a wide range of parameters, e.g., population density, age, and socio-economic status. By including small- to medium-size cities, such as Groningen, NL, and Swansea, UK, as well as large agglomerations like London, UK, and Toronto, CA, we can determine whether leak rates or emission characteristics differ or if systematic patterns emerge. Ideally, this joint analysis will help derive simple, generally applicable strategies for urban methane emission mitigation. We also demonstrate that a coherent data analysis methodology is important to allow intercity comparisons.

Section 2 briefly describes the cities under investigation, the general measurement principles, as well as the data analysis algorithm, before presenting and discussing the key results in Section 3. Key conclusions and recommendations for future work are given in Section 4.

## 2 Methods and Materials

### 2.1 City Descriptions

All cities included in this study are listed in Table 1. It is important to highlight that the mobile surveys did not strictly follow city or municipal limits. The exact definition of the (rectangular) area included for each city in our analysis is shown in Figure 2 (black frames), and the corner coordinates are given in Table S1. We also provide a short description of the total survey area, total population, and population density in Table 1.

**Table 1:** Information on Cities, Areas Surveyed, and Associated Population Based on the NASA SEDAC Population Estimation Service

city name	surveyed by laboratory # in year	description of main areas included in the surveys	land area included in the analysis (km^2^)	population in the analyzed area (million)	population density (thousands/km^2^)	pipeline material (PVC + PE/steel/cast iron/other)
(BAR) Barcelona, Spain	8 in 2018/2019	mostly within city limits	223	2.32	10.4	85%/13%/2%/0%
(BIR) Birmingham, United Kingdom	5 in 2019	urban region included	91	0.42	4.6	65%/6%/14%/16%
(BUC) Bucharest, Romania	3, 5 in 2019	some outskirts included	159	1.29	8.1	52%/48%/0%/0%
(GRO) Groningen, The Netherlands	6 in 2018	focus mostly on city of Groningen	73	0.14	2.0	80%/15%/3%/2%
(HAM) Hamburg, Germany	3 in 2018/2020	North Elbe	737	1.46	2.0	54%/44%/2%/0%
(KAT) Katowice, Poland	4 in 2018	surrounding urban areas included	793	1.29	1.6	40%/60%/0%/0%
(LON) London, United Kingdom	5 in 2018/2019	includes greater London metropolitan area	2029	9.74	4.8	65%/6%/14%/16%
(MUN) Munich, Germany	9 in 2018/2019	focus on city center	199	0.90	4.5	54%/44%/2%/0%
(PAR) Paris, France	7 in 2018/2019	includes Billancourt and Issy les Moulineaux	225	4.00	17.8	70%/26%/3%/1%
(SWA) Swansea, United Kingdom	5 in 2019	includes suburbs	123	0.19	1.6	65%/6%/14%/16%
(TOR) Toronto, Canada	1, 2 in 2018/2019	includes neighboring suburbs	562	2.25	4.0	N/A
(UTR) Utrecht, The Netherlands	3 in 2018/2019	inside the highway ring	48	0.16	3.4	80%/15%/3%/2%

**Figure 1:**
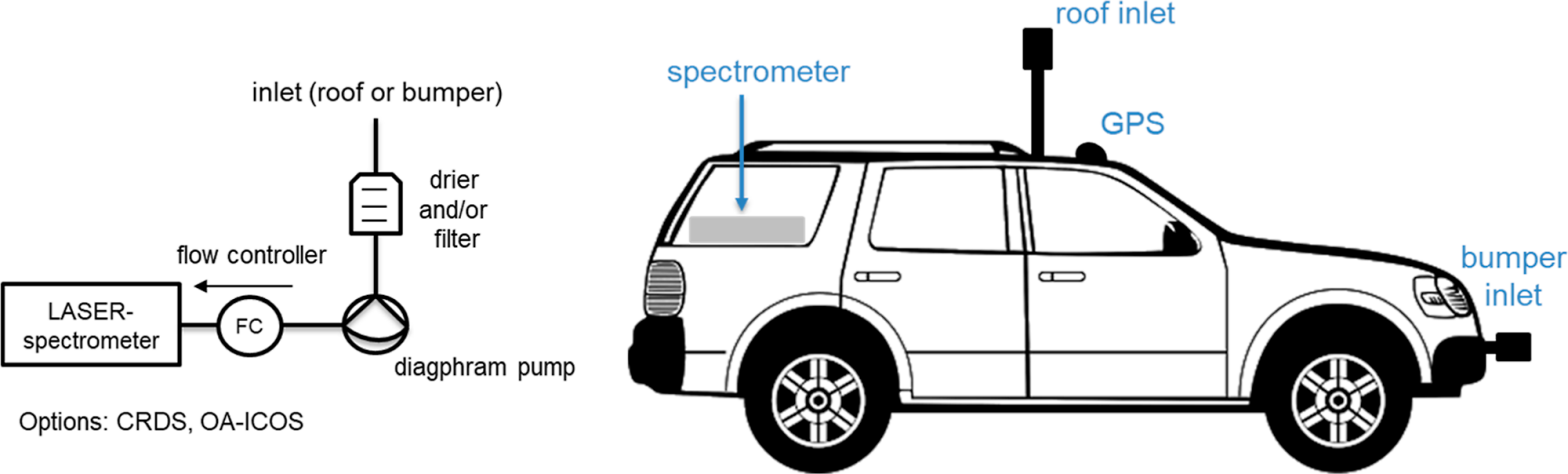
Schematic of typical measurement setup.

**Figure 2:**
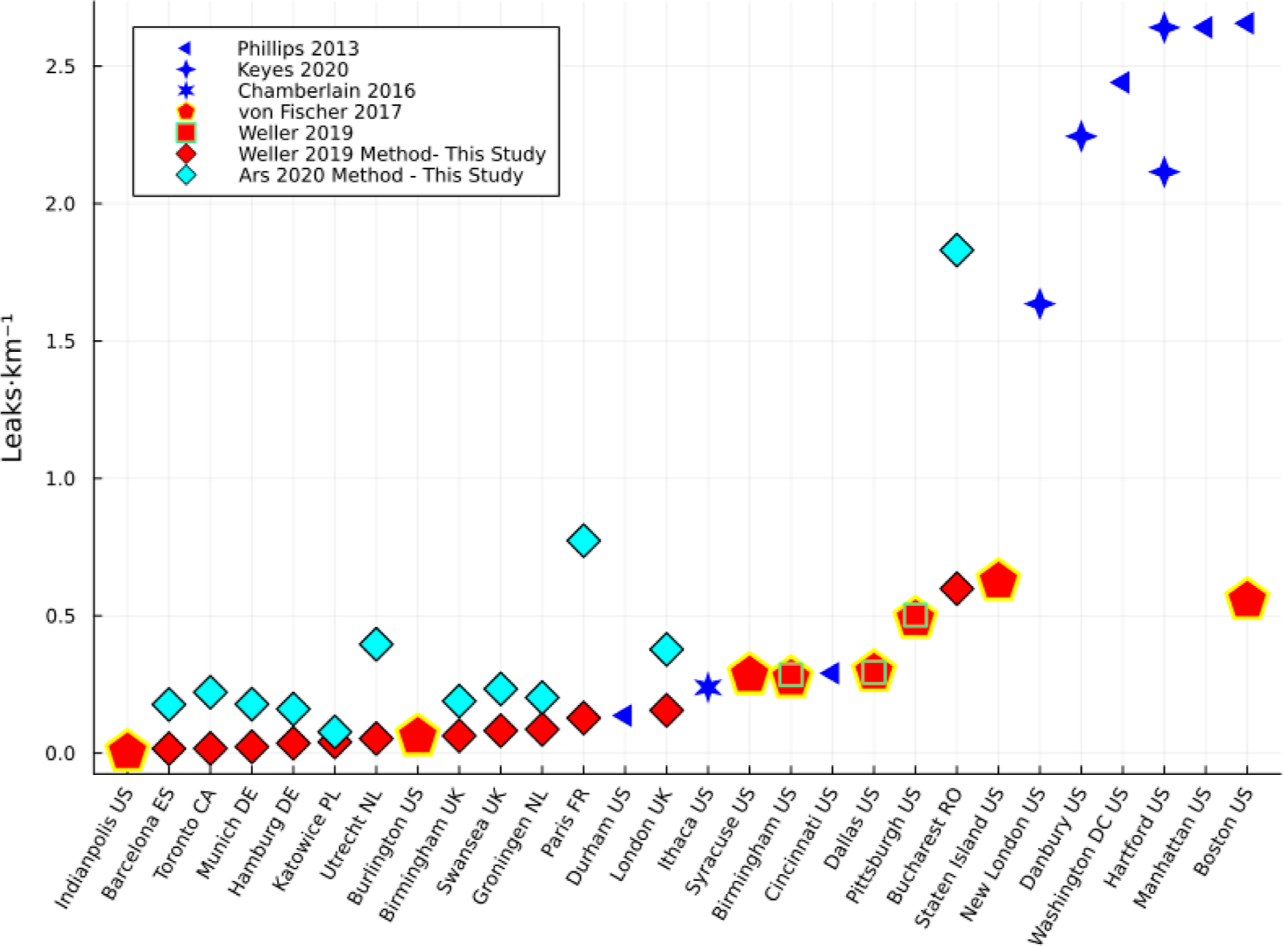
Leak indications per kilometer comparison across cities. The black-bordered rhombuses indicate cities from this study using two different classification methods (cyan: using Ars et al. 2020; red: using Weller et al. 2019). All other symbols reflect previous studies conducted in the US, with blue symbols indicating individual analysis methods and red symbols using similar methods, i.e., von Fischer et al. 2017 and Weller et al. 2019.

The population in each analyzed area is estimated using the NASA SEDAC population estimation service (https://sedac.ciesin.columbia.edu/). The area, population, and population density for the cities range over more than 1 order of magnitude. Pipeline material is reported according to Marcogaz’s national data sets, 2018.^14^ Our data set includes old cities, such as Paris and London, UK, and more recently developed cities, like Toronto, CA.

### 2.2 Measurement Principle

In each city, mobile platforms were deployed following the general schematic given in Figure 1. A high-precision fast-response greenhouse gas analyzer (see Table 2) is connected to an exterior inlet. Most frequently, the inlet was placed on top of the car, but in some cases, it was from the front bumper. Spatially referenced methane data was generated by combining the measured dry-air mole fractions of the mobile instrument with data from a synchronized GPS system. The spatial resolution of this mapping is limited by the frequency of the instrument’s measurements and the speed of the car. At typical speeds of 30–50 km/h and about 1–3 s to fully flush the measurement cells (cell turnover), this translates to a spatial resolution of about 10–40 m. A displacement correction was implemented to account for the inlet delay for each platform (see Table 2). Additional information on the mobile-based platforms for each city can be found in refs (9), (11), (12) and (15) or in the Supporting Information.

**Table 2:** Greenhouse Gas Analyzers and Other Instruments Used for Each City by the Local Survey Teams

city	GHG analyzer	inlet lag time (s)	GPS	inlet level
Barcelona, ES	G2301-m^a^	30	Garmin GPSmap	roof
Birmingham, UK; London, UK; Swansea, UK	G2301^a^/UMEA^b^	9/5	Hemisphere A21/Navilock-602	roof
Bucharest, RO	G2301^a^/G2401^a^/G4302^a^/UMEA^b^	17/18/5/10		roof/bumper
Hamburg, DE; Munich, DE; Utrecht, NL	G2301^a^/G4302^a^	3		roof/bumper
Groningen, NL	G2401-m^a^	5	Garmin Vivoactive3 sports	roof
Katowice, PL	G2201^a^/MGGA918^b^	7		roof
Paris, FR	G2401^a^/G2201-i^a^/MGGA^b^	20/30	Navilock-602U	roof/bumper
Toronto, CA	aG1301/aG2401	10	Airmar 220WX GPS	roof
Toronto, CA (bike)	UMEA^b^	30	Airmar 220WX GPS	mast (approximately roof height)

a Picarro INC, Santa Clara, USA.

b Los Gatos Research, San Jose, USA.

During this study, an alternative platform using a bicycle trailer was additionally used in Toronto. Fundamentally, the same concept was applied, by using an LGR ultraportable greenhouse gas analyzer (Los Gatos Research, now ABB, Zurich, Switzerland) in combination with an AIRMAR 220WX weather station (Airmar Technology Corp., Milford, US). The slower driving speeds and smaller vehicle allow access to narrower streets and detect weaker plumes, i.e., plumes with only small maximal methane enhancements. A more detailed description on the bike-based system and how it compares to car-based platforms can be found in Ars et al.^15^ All instruments used are of sub-ppm level precision and provide rapid cell turnover of 1–5 s, with the exception of the G2201 used in Katowice, PL, with a slower cell turnover of ca. 30 s.

### 2.3 Data Processing, Selection, and Emission Estimation

All measurement systems provided reliable localized methane mole fractions; however, multiple processing steps were needed to translate them into emission rate estimates. Here, we describe the different processing and quality control steps required to create a high-quality spatially explicit methane enhancement map. The algorithm used to translate this enhancement into an emission rate follows the principles of Weller et al.^8^

First, the raw data from the GHG analyzers was calibrated and combined with GPS data to create a synchronized data set, which was quality-controlled by the principal investigator (PI) for each city. Then, the data for each survey and city was analyzed to separate the local enhancements caused by CH_4_ emissions from variations in the atmospheric background CH_4_ mole fractions during the surveys. In principle, a moving window fit was used to determine the typical variability of the background and situations when mole fractions exceeded this threshold. A detailed description of the algorithm and the reference to the source code is provided in Ars et al.^15^

After removing the background, we then identify the individual enhancements from local emissions. Previous studies have shown that plumes from natural gas distribution leaks are typically highly localized, which is why broad plumes were not considered further. Following Weller et al.^8^ and Maazallahi et al.,^9^ we chose a cutoff of 160 m for the maximum plume width. In the next processing step, the remaining enhancements are then mapped; that is, plumes that overlap or fall within 50 m of each other are considered to originate from the same source. When multiple plumes were observed during the same survey, i.e., typically within a few minutes at the same location, the maximum value is selected as the most appropriate. On such short time scales, it is unlikely that the emission rate has changed significantly, but we know that local wind patterns can vary on such short time-scales. Hence, selecting the maximum of the data from the plume crossing with the maximum enhancements ensures that we are not artificially biasing our data low by combining situations where the full plume was captured (i.e., maximum peak height and area observed) with situations where wind conditions were suboptimal and part of the plume was missed. An important next step was to identify any plumes from sources that are not associated with natural gas. To achieve this, we compared the list of plume locations with a list of known methane sources: e.g., landfills, sewage systems, or dairy farms for each city. Some PIs also provided information about the isotopic composition in each plume and whether it was consistent with thermogenic methane sources. As ethane is frequently present in natural gas, it was another useful proxy which we used wherever possible. Another filtering step used to classify plumes was their persistence. If plumes were observed several times at the same location, we assume that a local source was responsible.

Finally, after all processing and selection steps were applied, we now considered the remaining methane plumes as leak indications related to urban natural gas infrastructure.

These leak indications were then converted into local emission rate estimates. As complex atmospheric modeling is not possible for the hundreds or thousands of leak indications, we relied on the previously used and tested empirical equation suggested by Weller et al.^8^
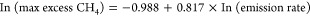
1where the emission rate is given in L min^–1^ and the maximum CH_4_ excess above the background in ppm, seen in the plume crossing. Weller et al.^16^ calibrated this equation using controlled release experiments under idealized conditions for methane emission rates, ranging from about 0.5 L to 55 L min^–1^ and at distances of 0–80 m downwind of a known source. Unfortunately, no rigorous uncertainty calculation was performed in this study. However, we know that using different equipment or inlet setup can cause over- or underestimation of emissions; for example, a 10% underestimation of the local methane enhancement can lead to a 12% underestimation of the local emission rate (see Supporting Information S4).

We note that several of the city surveys that are evaluated and compared here represent snapshots where the goal was to cover large parts of a city in a relatively short period. A disadvantage of this approach is that many streets were surveyed only once or twice. Controlled release experiments^7,16^ and frequent passages of the same leak location^17−19^ have shown that emission estimates from a single detected source can vary by more than an order of magnitude between individual passages and that emission rate estimates based on infrequent visits are biased high. A simple explanation for changing local enhancements is changes in wind speed and direction. Higher wind speeds will typically disperse the local methane plumes more rapidly and, hence, dilute the locally detectable signal. Furthermore, infrequent visits might lead to missing leak indications if surveys are only conducted when leak sites are downwind of the surveyed road. The consequences for our study are discussed below.

### 2.4 Summary of Data Analysis Methodologies in Other Urban Methane Surveys

In the available literature, seven studies estimate natural gas leak occurrence rates from 16 different U.S. cities surveyed using vehicle-based mobile laboratories equipped with Picarro CRDS instruments.^7,8,20−24^ Of these studies, four use absolute observed methane mole fractions as a metric for leak indications. Studies following the Phillips 2013 methodology classify leaks as methane mole fractions exceeding 2.5 ppm, with peaks within 5 m binned together.^20−22^ Chamberlain et al. from their surveys in Ithaca, NY, US, classified leaks as concentrations in excess of 1.93 ppm.^23^ Von Fischer et al. and Weller et al. developed statistical algorithms for quantifying emissions from observed concentration enhancements above a background and bin observations within 30 m together.^7,8^ Among their results from five cities, von Fischer et al., significantly, present results from Boston, MA, US, the first city surveyed using the Phillips 2013 method. The von Fischer 2017 method found vastly fewer different leaks per kilometer, which they reconciled to be a consequence of the difference in spatial binning between the two methods.^7,22^ The von Fischer 2017 and Weller 2019 methods, analyzed across four cities, were found to return similar leak counts.^8^ Another statistical method, based on a modified Tau approach which identified outlying CH_4_ observations, used 30 m binning for observed peaks for three cities in Connecticut, US, and found a similar rate of leaks per mile to the Phillips 2013 methodology in cities with similar infrastructure to Boston, MA, US.^24^ One study analyzed data from a mobile laboratory in Los Angeles, CA, US, but averaged data across 5 s and binned data along 150 m long road segments in order to analyze CH_4_/C_2_H_6_ ratios to evaluate the contribution of natural gas to measured CH_4_ enhancements.^25^ Similarly, mobile mapping was conducted in Indianapolis, IN, US, with a primary focus on measuring known point sources in the city.^26^ Lastly, a study from Beijing, China, has also presented CH_4_ survey results, with a primary focus on determining CO_2_/CH_4_ emission ratios from known CH_4_ sources.^24^ Leak occurrences were not calculated in these studies. Additional information on methods can be found in Section S3 in the Supporting Information.

### 2.5 Inventory-Based Estimates of City-Wide Methane Emissions from Natural Gas Infrastructure

Two different inventory-based estimates are used for comparison with the observation-based estimate. Our inventory-based estimate uses National Inventory Report (NIR) data and the CRF (Common Reporting Format) tables for the natural gas distribution sector (i.e., sector 1B2b5) for 2018, as available at the UNFCCC website (https://unfccc.int). The national emission estimate is downscaled to the respective cities based on national population density for the area covered using LandScan gridded population data for 2015.^27^ The national inventories of different countries may use different methods to estimate methane emissions from gas distribution, as described in their NIR (https://unfccc.int/ghg-inventories-annex-i-parties/2020). An alternative methodology is published by Marcogaz^14^ and uses gas distribution network length, network (pipeline) material, pipeline material-specific emission factors, and the number of connection points and city gates to estimate methane emissions. Methane emission from total national gas distribution networks have been calculated using the emission factors and gas distribution network data presented in Marcogaz^14^ and subsequently downscaled to each city based on population data,^19^ similar to the procedure for the NIR CRF emission estimates. The table with city-specific values can be found in Section S5.

## 3 Results and Discussion

In this chapter, we present the leak indication statistics for all cities in Section 3.1 and the comparison between emission rate estimates and inventory-derived estimates in Section 3.2, and we assess the influence of large emitters on citywide natural gas-related emissions in Section 3.3.

### 3.1 Statistics and Mapping of Leak Indications

Previously published studies using mobile surveys to quantify methane from natural gas infrastructure varied slightly in their setup and data treatment; thus, the focus of this study is to process and analyze data from all 12 cities consistently (see Section 2.3) such that they are directly comparable with some previous studies conducted in the US (e.g., refs (7), (8) and (16)). We present results collected by surveying over 20,000 km of roads with about 10,000 km of unique road segments visited between 1 and 10 times during the different survey periods.

The leak indications presented in Table 3 are classified into four categories based on the maximum methane enhancement found in the plume, i.e., the maximum concentration minus the background (see Section 2.3). The limits of high (≥7.6 ppm), medium (7.59–1.6 ppm), low (1.59–0.2 ppm), and lowest (0.19–0.04 ppm) plume mole fractions were chosen. The first three categories are consistent with previous studies conducted in US cities (e.g., ref (16)), while we added the lowest category based on findings by Ars et al.^15^ that were able to confirm smaller leaks using a bike-based survey system. We also report leak indication rates per kilometer, (1) following Weller et al.,^16^ which only accounts for the largest three peak categories, and (2) following Ars et al.,^15^ which reports higher rates due to the inclusion of the lowest category of peaks. This lowest category of peaks, i.e., 0.19–0.04 ppm, was previously only reported by Ars et al.^15^ and not included in studies for other cities. For the 12 cities in this study, we find that the leak rates differ significantly from city to city, with the highest rates found in Bucharest, RO, and London, UK, while Munich, DE, Hamburg, DE, and Barcelona, ES, are found on the lower end for both of the leak rates for both analysis techniques. In general, one would expect that the pipeline infrastructure predicts emission rates, and as seen in Table 1, Barcelona, ES, is likely to have the highest percentage of PE&PVC pipelines, while UK cities have a significantly higher share of cast iron or other materials, which are known to be more leak-prone.

**Table 3:** Statistics from Surveys on Methane Enhancements from Natural Gas Sources Found in Each City^a^

city name	high CH_4_ enhancement	medium CH_4_ enhancement	low CH_4_ enhancement	lowest CH_4_ enhancement	leak indications per 100 km calculated following Weller et al.^8^ (only accounting for high, medium, and low enhancements)	leak indications per 100 km calculated following Ars et al.^15^ (including the lowest enhancement category)	roads covered (km)	total roads within domain (km)
	≥7.6 ppm	7.59 to 1.6 ppm	1.59 to 0.2 ppm	0.19 to 0.04 ppm				
Barcelona, Spain	0	1	5	59	2	18	367	2171
Birmingham, United Kingdom	0	1	12	26	6	19	206	1121
Bucharest, Romania	8	36	384	881	60	183	715	2086
Groningen, The Netherlands	1	5	18	32	9	20	277	709
Hamburg, Germany	0	5	52	196	3	14	1576	4617
Katowice, Poland	0	5	38	39	4	8	1062	3889
London, United Kingdom	4	65	494	802	16	38	3615	18,397
Munich, Germany	0	1	2	20	2	18	129	2081
Paris, France	0	3	59	313	13	77	485	3394
Swansea, United Kingdom	0	3	17	37	8	23	244	745
Toronto, Canada	0	1	18	227	2	22	1110	5750
Utrecht, The Netherlands	1	5	19	162	5	40	472	757

a Unique road kilometers covered calculated from GPS data and total road kilometers within domain from OpenStreetMap data. The leak indications per 100 km are reported as a range, with the lower end only accounting for large, medium, and small peaks (as common in previous US studies), while the upper end of the range includes the lowest peak category as well, as introduced by Ars et al.^15^

Unfortunately, city-specific pipeline data is not publicly available to explain the ranking of each individual city as seen in Figure 2. Furthermore, discrepancy in leak indication rankings for cities like Utrecht, NL, becomes difficult when comparing the two different peak classification techniques. As expected, Ars et al.^15^ reports more frequent leak indications. Especially Paris, FR, and Bucharest, RO, report a lot of peaks in the lowest category, while Katowice, PL, does not display many additional leaks. However, this can easily be explained as the instrument used for surveys in Katowice, PL, was a slower-response instrument which fails to detect smaller local methane enhancements; hence, using the Ars et al. method does not add a lot of leak indications in the lowest category. For Bucharest, RO, and Paris, FR, previous work suggested that potential sewage-related sources could exist,^11,12^ and recent work in Montreal^28^ confirmed that sewage-related methane emissions are typically 1 order of magnitude smaller than oil- and gas-related emissions. Hence, the lowest emission category could likely include such sources more commonly; hence, focus on the Weller-based results will be given here. For other cities in this study, we do not expect a significant impact from the experimental setup as fast instruments, even when used at the bumper versus the roof, will agree within ca. 10–30% in their local enhancements and emission rates (see supplementary Section S3 and Ars et al).^15^

When comparing our data set to the previous studies conducted in the US, the issue of different methodologies becomes even more apparent. Unfortunately, US surveys initially used bespoke analysis methods, i.e., differences in how local enhancements are calculated and how they were translated into local emissions (see Section 2.4). Figure 2 illustrates how methodological differences complicate intercity comparison. Especially, the case of Boston highlights the impact of different methodologies and surveys in independent studies as the reported leak rate differs by a factor of 5. For our comparison, we focus on the data sets for our 12 cities and previous work using Weller al.^16^ Despite the same analysis technique, differences between cities in the US and most European cities are evident. Most of the European cities are close to the cleanest US cities, i.e., Indianapolis, IN, US, and Burlington, CT, US, with the exception of Bucharest, RO, which is comparable to highly emitting US cities like Staten Island, NY, US, and Boston, MA, US.

As an additional caveat for the data collected in our 12 cities, some of the high emission rate estimates might be the result of poor sampling statistics for these locations, as a consequence of the strategy of short-term wide-area surveys with infrequent revisits (e.g., Munich, DE, and Birmingham, UK), which can lead to an overall overestimate in the emission rate.^18^ We note that cities that have been surveyed more completely and frequently (e.g., Toronto, CA, and London, UK) should be less susceptible to these high biases, because unusually high local methane enhancements that can lead to very high emission rate estimates will average out when combined with data from multiple survey days. In contrast to this expectation, the leak rates found in this study appear to be independent of the length of roads surveyed or percentage of road covered in each city, highlighting that leak indications are likely not a feature of the sampling density here.

**Figure 3:**
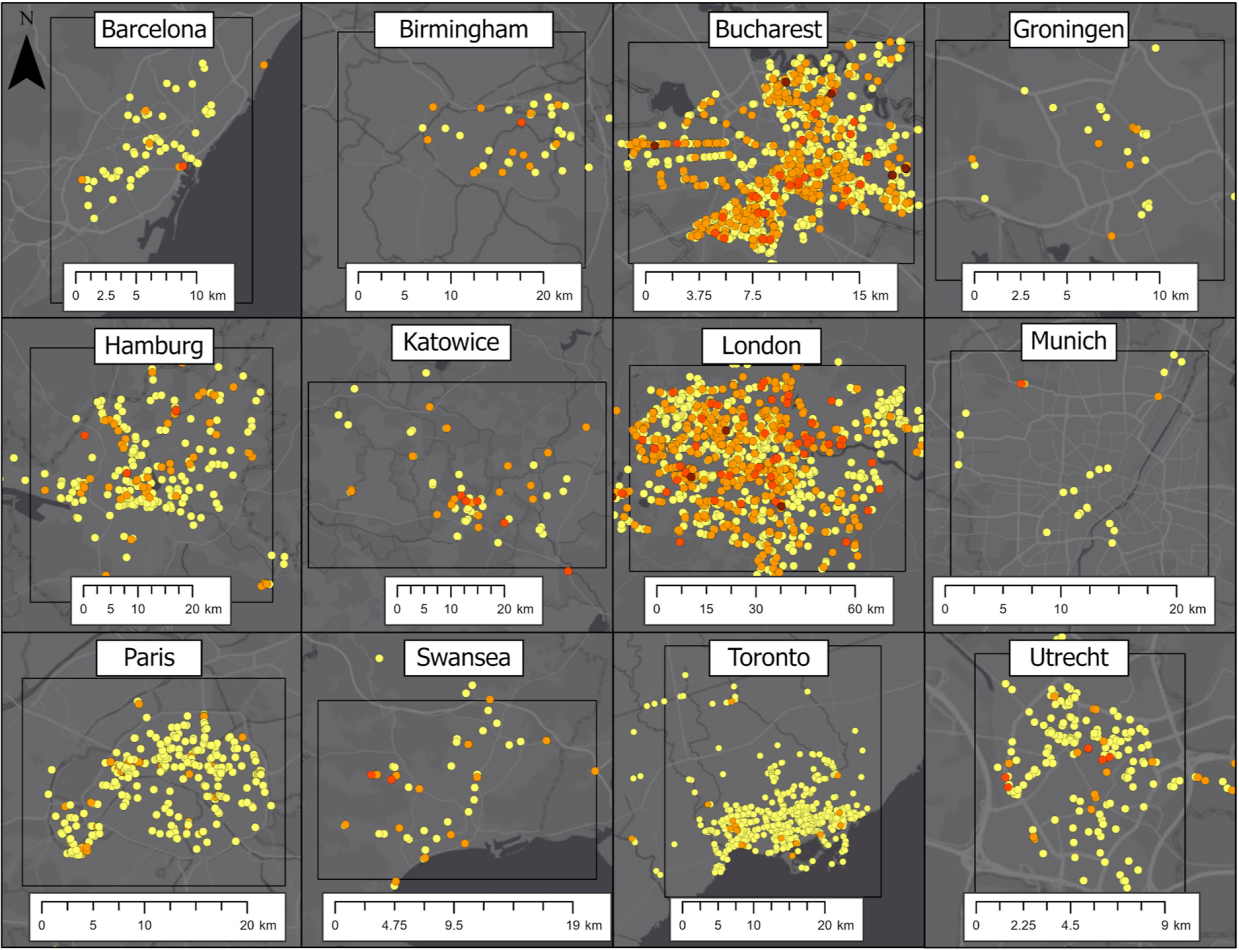
Methane leak indication map for each city categorized by maximum enhancement above the background; yellow: lowest (0.04–0.19 ppm), orange: low (0.2–1.59 ppm), red: medium (1.6–7.59 ppm), and dark red: high (≥7.6 ppm). The city domain included in the analysis is represented by the black frame. Note that cities are displayed on differing spatial scales for convenience.

We furthermore find that despite small differences in the evaluation, the ranges of leak indications in this study agree generally with previous studies published on Toronto, Hamburg, DE, Utrecht, NL, and Paris, FR.^9−12,15^ We created maps for each city to visualize the spatial patterns of emissions (see Figure 3), and it is apparent that while very low enhancements can be found in many locations, medium and larger enhancements are rarer in all cities compared to those reported in the single-city papers. The maps created here were compared with existing maps for previously published work in Hamburg, DE, Utrecht, NL,^9^ and Paris, FR,^11^ which use slightly different analysis methods. We find minor differences, which can be attributed to differences in the definition of the background, peak selection based on plume width, or minimum distance from non-natural gas methane sources. In this study, we focus on the systematic differences in city-scale emissions observed when using the same (consistent) analysis algorithm everywhere. The leak locations can be seen in Figure 3.

### 3.2 Comparing Observation-Based Emission Estimates to Inventory Data

**Figure 4:**
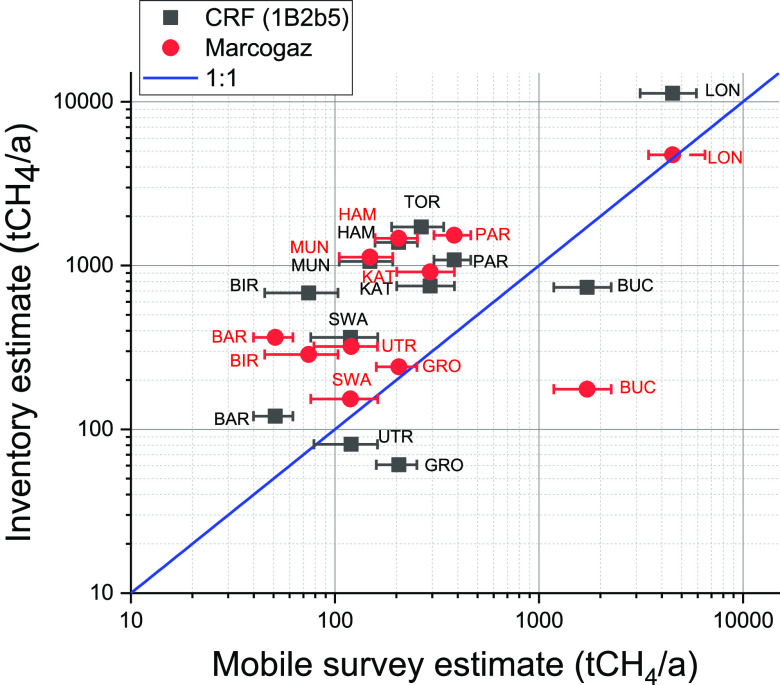
Comparison of citywide methane emissions from natural gas distribution derived from mobile surveys and inventory estimates based on (a) Marcogaz (2018) emission factor and pipeline data (red circles) and (b) downscaled UNFCCC national inventory data in common reporting format (black squares). Definitions for the three-letter city abbreviations can be found in Table 1.

To compare the mobile survey data to inventory estimates, we scaled up the emission estimates from the roads surveyed to the whole city. To achieve this, the emissions from the roads surveyed are scaled to match the total road network length for each city domain, which was calculated from OpenStreetMaps road data (Table 3). The underlying assumption is that in a given city, 1 km of roadway roughly equates to 1 km of pipeline, which seems reasonable in residential areas, where natural gas is used in most parts of the city for energy or cooking. For transit roads, this might not hold true, but this scaling approach was successfully used in previous studies (e.g., refs (11) and (15)). Note, however, that Maazallahi et al.^9^ showed that emissions between residential roads and larger streets can differ. To understand the relationship of road kilometers and pipeline infrastructure, we analyzed data available for Paris, FR, Munich, DE, Bucharest, RO, and Katowice, PL, and found that the total road length is similar to the length of distribution pipelines to within 20–30%. This uncertainty in upscaling is included in our citywide emission estimate. Additionally, we provide a lower bound estimate of the emissions by excluding the category of “lowest peaks”, which also slightly increases the uncertainty range. Lastly, we expect an uncertainty of 20–30% of leak enhancements depending on instrument inlet location and instrument response time (see Supporting Information S4). As city-specific methane inventories only exist for a small number of cities (e.g., refs (26) and (29)), we compare our observation-based estimate with two scaled inventory estimates. The CRF estimate uses NIR data for the natural gas distribution sector (i.e., 1B2b5) downscaled to each city based on population data, while the Marcogaz estimate uses national natural gas consumption data and per capita emission factors (see Section 2.4). Due to the differences in city size, infrastructure, and energy consumption, we find that the citywide emissions estimates span multiple orders of magnitude (see Figure 4). The observed and inventory estimates over all the cities correlated with an *R*^2^ accounting for errors y dimension of 0.74 and 0.89 for the Marcogaz and CRF estimate, respectively. We also find that almost all the cities lie above the 1:1 line, indicating that observed emissions are lower than what would be predicted based on consumption or national inventory reporting data for the whole natural gas distribution sector. This should be expected as our surveys excluded emissions from large facilities, such as transmission, compressor, feeder, and industrial metering stations. Nonetheless, our study suggests that a very significant share of emissions in this sector can be directly detected and assessed using mobile surveys. There are unusually high emissions reported for Bucharest, RO; however, recent work by Fernandez et al.^12^ indicates that roadside methane plumes are more common in Bucharest, RO, due to significant contributions from the local sewers and wastewater collection system. This additional source would also explain why Bucharest, RO, shows a very high number of peaks in the lowest category as sewage emissions tend to be significantly lower than O&G related sources in an urban environment.^28^ It is also apparent that significant discrepancies between the inventory estimates themselves and the observation-based estimates exist for individual cities.

### 3.3 Importance of Large Emitters

**Figure 5:**
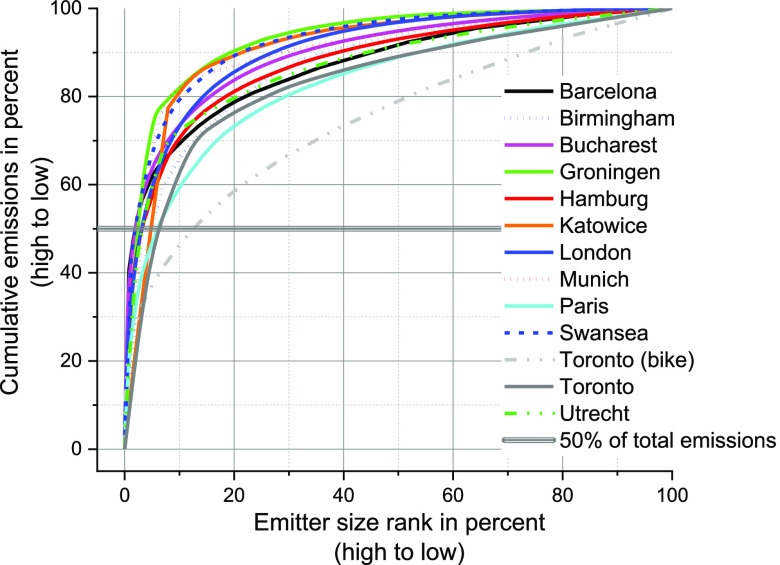
Normalized cumulative emissions relative to normalized emitter size ranked from high to low emissions for each city.

As reported in Section 3.1, most of the plumes associated with methane leak indications fall into the smaller categories. Nevertheless, even a few large leaks can significantly influence the citywide emission rate given the nonlinear relationship between observed plume enhancement and emission rate estimates (see eq 1 in Section 2.3). To investigate the impact of large emitters for each city, we rank leak indications by their emission rate (high to low) and calculate how much they cumulatively contribute to the overall emissions. As the results of overall emissions span a wide range for the 12 cities, we normalize the emissions for each city to 100% (see Figure 5). Despite slight differences between cities, a clear pattern emerges where the top 10% of emitters are typically responsible for 60–80% of citywide natural gas distribution-related emissions. Overall, these distributions agree qualitatively with the heavy-tailed distributions found for cities in the US^8,30^ despite the difference in the survey methodologies that caused the visible difference for the leak rates. Even for the bike-platform used in Toronto, CA, which is tailored to finding many more enhancements in the lowest category, the highest 17% of emitters contribute 50% of total emissions. A possible caveat of the ranked evaluation is the high uncertainty of emission rate estimates from infrequent revisits, which may overemphasize the highest emitters during very short-time surveys, while cities with frequent revisits like Toronto, CA, and London, UK, will have the most reliable results. Similar to the findings in Section 3.2, our study seems not to be qualitatively biased by sampling frequency as even cities like Munich, DE, with very limited coverage, display this heavy-tail behavior.

## 4 Implications and Limitations

The successful deployment of mobile survey platforms across 12 cities within this project, funded by the Climate & Clean Air Coalition, highlights that scientifically robust data on natural gas distribution-related emissions based on observations can be collected rapidly by academic and government organizations.

The relatively lower emissions from European and Canadian cities compared with US cities^8,16,17,20−24,31^ further highlights that cities with excessive methane emissions should act rapidly. The difference in emissions is most likely due to differences in local infrastructure and, particularly, pipeline materials. Furthermore, natural gas infrastructure is more tightly regulated in the European Union than in the US. We infer large differences in citywide emission estimates between the cities we have analyzed so far, which underlines that more cities should be investigated in the future to find major emitters in as many places as possible.

From a global point of view, novel portable spectrometers facilitate wide deployment, and the workflow and processing algorithms are publicly available. This should make it easier to conduct extensive surveys elsewhere in support of local mitigation efforts. It seems therefore logical to expand these studies from North America and Europe to other global regions to investigate whether emission patterns are similar to those found here, especially in developing economies. Our analysis suggests that the best strategy is to start with cities and regions with older or vulnerable infrastructure and identify the strongest emitters. It also seems crucial to revisit cities on a regular basis to ensure that exceptionally strong emission sources have been mitigated and to ensure that new emerging sources are added to mitigation activities.^21^ To achieve such a comprehensive monitoring program, it will be necessary to move beyond studies that are driven by academic research groups and to invite the participation of government agencies and/or private sector actors. Good examples of how existing infrastructure could be used for regular surveys of urban greenhouse gas levels are presented in the study by Mallia et al.,^32^ where greenhouse gases are surveyed multiple times a day across the city using a tram line, and in the study by Weller et al.,^8^ where Google Street View vehicles were used as a mobile platform.

Beyond these findings, our study demonstrates the value of analyzing different data sets in a common manner if internally consistent, multicity emission comparisons are to be achieved in the future. The logical next step would be to work toward a standardized approach for mobile monitoring. It is apparent from this work that an upgraded empirical equation to better fit various types of natural gas infrastructure in other regions of the globe and robust quantification of minor enhancements are needed to (a) reduce uncertainties and (b) better quantify emissions from smaller sources, which can be detected by slower platforms, as demonstrated by the bike-based system.

A standardized approach deployed on equivalent mobile platforms holds the promise to rapidly decrease urban methane emissions from the natural gas infrastructure while also providing data to the academic community to further investigate the impact of other urban sources of methane, e.g., furnaces, sewage systems, and other natural systems, which are currently not well constrained.

## Data Availability

Processed and raw data are available through ECCC’s data server at https://crd-data-donnees-rdc.ec.gc.ca/CCMR/publications/2024_Vogel_ES&T_TwelveCitiesMethane Codes for data processing are available on github at https://github.com/WunchLab/.

## Supporting Information Available

Description of the measurement platform for each city; definition of urban domains; review of urban methane surveys in the US; uncertainty estimation of the inlet/sampling system and other factors; additional information used for bottom—up emission estimates; and pipeline material information (PDF)
