# Supplementary material for: Ground-Based Mobile Measurements to Track Urban Methane Emissions from Natural Gas in 12 Cities across Eight Countries
Source: Environ Sci Technol. 2024 Jan 25;58(5):2271–81. doi: 10.1021/acs.est.3c03160 (PMC10851421; doi:10.1021/acs.est.3c03160)
Supplement: Supplementary file 1 — es3c03160_si_001.pdf [file es3c03160_si_001.pdf]

Supplemental materials for "Ground-based mobile measurements to track urban methane emissions from natural gas in twelve cities across eight countries".

F. Vogel[1], S. Ars[1], D. Wunch[2], J. Lavoie[2], L. Gillespie[1,2], H. Maazallahi[3], T. Röckmann[3], J. Nęcki[4], J. Bartyzel[4], P. Jagoda[4], D. Lowry[5], J. France[5,16], J. Fernandez[5,17], S. Bakkaloglu[5,18], R. Fisher[5], M. Lanoiselle[5], H. Chen[6,19], M. Oudshoorn[6], C. Yver-Kwok[7], S. Defratyka[7,20], JA. Morgui[8], C. Estruch[9], R. Curcoll[8,10], C. Grossi[10], J. Chen[11], F. Dietrich[11], A. Forstmaier[11], H.A.C. Denier van der Gon[12], S.N.C. Dellaert[12], J. Salo[13], M. Corbu[14,15], S.S. Iancu[14,15], A.S. Tudor[14,15], A.I. Scarlat[14,15], A. Calcan[15,21]

1 Climate Research Division, Environment and Climate Change Canada, Toronto, M3H 5T4, Canada
2 Department of Physics, University of Toronto, Toronto, M5S 1A7, Canada
3 Institute for Marine and Atmospheric Research Utrecht, Utrecht University, Utrecht, 3584 CC, The Netherlands
4 AGH, University of Kraków, Kraków, 30-059, Poland
5 Department of Earth Sciences, Royal Holloway University of London, Egham, Surrey, TW20 0EX, United Kingdom
6 Centre for Isotope Research, Energy and Sustainability Research Institute, University of Groningen, Groningen, 9747 AG, Netherlands
7 LSCE, CEA-CNRS-UVSQ, University Paris-Saclay, Gif-sur-Yvette, 91191, France
8 ICTA, Autonomous University of Barcelona, Barcelona, 08193, Spain
9 Eurecat, Centre Tecnològic de Catalunya, Barcelona, 08290, Spain
10 INTE, Universitat Politècnica de Catalunya, Barcelona, 08028, Spain
11 Environmental Sensing and Modelling, Technical University of Munich, Munich, 80333, Germany
12 Netherlands Organisation for Applied Scientific Research - TNO, Utrecht, 3584CB, The Netherlands
13 Geography and GIS, University of Northern Colorado, Greeley, Colorado, 80639, United States
14 Faculty of Physics, University of Bucharest, Bucharest, 050663, Romania
15 INCAS, National Institute for Aerospace Research "Elie Carafoli", Bucharest, 061126, Romania
16 now at: Environmental Defense Fund, 41 Eastcheap, London, EC3M 1DT, United Kingdom & Royal Holloway University of London, Egham, Surrey, TW20 0EX, United Kingdom
17 now at: University of Maryland, College Park, Maryland, 20742, United States
18 now at: Sustainable Gas Institute, Imperial College London, London SW7 2AZ, United Kingdom
19 now at: Joint International Research Laboratories of Atmospheric and Earth System Sciences, School of Atmospheric Sciences Nanjing University, Nanjing, 210023, China
20 now at: University of Edinburgh, Edinburgh, EH8 9YL, United Kingdom and National Physical Laboratories, Teddington, TW11 0LWUnited Kingdom
21 now at: United Nations Environment Programme, Paris, 75009, France

Summary: 10 pages, 1 figure, 3 tables

S1. Description of measurement systems

In this section, a more detail description of the measurement setups and field campaigns for each city is given.

Barcelona, Spain:

The mobile surveys were conducted in Barcelona, ES between December 2018 and September 2019. The surveys were conducted using a Citroen Picasso, with the measuring instruments located in its trunk. The inlet was located in the car roof rack with a methacrylate protection to avoid water and dust entering the line. A GPS (Garmin GPSmap) and a Kestrel 5500 sensor for temperature, pressure and humidity were collocated at the car roof rack.

Synflex tubbing of ¼" was used to connect the inlet with the analyzer. A 'flight ready' Cavity-Ring Down Spectroscopy based analyzer measuring CO_2, CH_4 and H_2O (Picarro G2301m) was used for the surveys. The sampling frequency was 1Hz for both GPS and Picarro instruments. A delay time of 30 seconds was observed for the inlet. In every survey, the Picarro time was synchronized with the GPS UTC time. An APC uninterruptible power supply with 5 additional battery packs was used to guarantee a run time of 6 hours for the G2301m and its associated vacuum pump.

The G2301m was regularly calibrated for CO_2 and CH_4 each two months using 6 NOAA primary standards with a linear fit. The CO_2 and CH_4 values were also corrected for water concentration using the CO_{2_wet}/CO_{2_dry} and CH_{4_wet}/CH_{4_dry} fit values obtained from a previous water calibration done with a dew point generator.

Birmingham, United Kingdom:

Surveys of Birmingham started January 2019 and the 5 days of surveys were completed by the end of October 2019. The area surveyed is bounded by the M5 motorway in the west, the M42 motorway in the south and east, the M6 Toll road in the NE, and the A4041 road in the NW. For mobile measurement setup see descriptions for London, UK.

Bucharest, Romania:

The Bucharest, RO campaign was conducted in the late summer 2019. 9 surveys were carried out between the 20th and 29th of August, that utilized 3 vehicles and 4 different CRDS instruments. Three Picarro instruments were used: model G2301 (CH4, CO2, and H2O), model G2401 (CH4, CO, CO2, and H2O), and a Gas Scouter TM model G4302 (CH4, C2H6, and H2O) (see Mazallahi et al. (2020) for details on the G2301 and G4302). A Los Gatos Research Ultraportable Methane Ethane Analyzer (LGRUMEA) was also used (see London, UK for details).

The three vehicles were equipped with an additional external battery to power the instruments. An external sampling inlet was installed into each vehicle which led from the front bumper to the interior of

the rear trunk, and was connected to an instrument intake, or a split to connect to more than one instrument. The front bumper inlet was 60 cm from the ground level of each car. Inlet delays were recorded daily and averaged as follows, G2301 at 17 s, G2401 at 18 s, G4302 at 5 s, and the LGRUMEA at 10 s. Each vehicle had a GPS unit and anemometer measuring location coordinates, windspeed, and direction every second. Measurements were displayed either with a netbook, tablet, or monitor via internal WiFi with ethernet or virtual network computing (VNC) connection.

All 4 instruments measured 4 known mixtures of standard gases ranging from ambient Methane mole fractions to 130 ppm CH4, both before the start of the campaign and the end. This produced a 4-point calibration curve which was later applied to correct the data. At the start of the day, each vehicle team was assigned a specific region of the city with a target distance to be covered. For matching of data and measurements, instruments and measuring equipment were synchronized at the start of the day to UTC (+2). All surveys were about 6 to 8 hours in duration and were carried out during daylight hours to avoid low boundary layer conditions.

Groningen, The Netherlands:

The mobile measurements were made using a Picarro mobile cavity ring-down spectrometer (2401-m). During the measurements, the Picarro was located in a car connected to a vacuum pump and was powered by a large battery pack. The battery pack consists of three 12V 115Ah units.

An ADM100 flow meter from Agilent was used to measure and check the flow through the system. During all days, the flow rate was constant. The flow rate of the system was ~265 ml/min, combined with a tube of 420 cm with an inner diameter of 4.2 mm. Mass flow calculations resulted in a lag of 5.4s.

The ¼" inlet line is connected to a mast mounted on the roof of the car for continuous sampling of ambient air at a height of 220cm above ground level. The distance between the rooftop and the inlet line is about 20cm to avoid measuring the boundary layer from the car. No filters were used in front of the inlet line. For the GPS, a Garmin Vivoactive 3 sports watch was used.

Meteorological data is retrieved from the KNMI (Royal Netherlands Meteorological Institute) for the measurement station at the Groningen, NL Airport. When a large plume was detected and the road conditions allowed us to stop, we were able to measure wind speed and wind direction with a handheld Kestrel 5500 wind meter. The choice for a handheld meter was made for simplicity reasons.

During the measurements, an average driving speed in the range of 25 – 30 km/h was used. If an enhanced concentration was found and safety allowed, lower speeds were used to re-drive the same area or even stop.

Two measurement campaigns have been conducted during this project, with a total of 10 measurement days. The first campaign was conducted on 6,7,8 August 2018. The second campaign took place between 25 September – 3 October. During both campaigns, the city of Groningen, NL has been mapped using the mobile laboratory. A total of 909.2 km was travelled for the measurements, of which 515.7 km in the city of Groningen, NL and 393.5 km in the province.

Hamburg, Germany:

Mobile atmospheric measurements at street level were conducted using two Cavity Ring-Down Spectroscopy (CRDS) analyzers (Picarro Inc. model G2301 and G4302). The model G2301 instrument

provides atmospheric mole fraction measurements of CO_2, CH_4 and H_2O, each of them with an integration time of about 1 sec., which results in a data frequency of ≈ 0.3 Hz for each species. The reproducibility for CH_4 measurements was ≈ 1 ppb for 1 s integration time. The G2301 instrument was powered by a 12 V car battery via a DC/AC converter. The flow rate was ≈187 ml/min. Given the volume and pressure of the measurement cell (volume = 50 ml and pressure ≈ 190 mbar) the cell is flushed approximately every 3 s, so observed enhancements are considerably smoothed out.

The G4302 instrument is a mobile analyzer and provides atmospheric mole fraction measurements of C_2H_6, CH_4 and H_2O at ≈ 1 Hz frequency at a flow rate of ≈ 2.2 L/min using an interior pump with cavity size of 35 ml at controlled pressure of 600 mbar. The G4302 runs on a built-in battery which lasts for about ≈ 6 h. The instrument can be operated in two modes at ≈ 1 Hz frequency for each species: the CH_4-only mode and the CH_4 - C_2H_6 mode. In the CH_4-only mode the instrument has a reproducibility of ≈10 ppb for CH_4. In the CH_4 - C_2H_6 mode the reproducibility is about 100 ppb for CH_4 and 15 ppb for C_2H_6. For Utrecht, NL surveys, the G4302 was not yet available for the initial surveys in 2018, but it was added for the later re-visits. For Hamburg, DE, both instruments operated during the entire intensive 3-week measurement campaign in Oct/Nov 2018. The time delay from the inlet to the instruments was measured and accounted for in the data processing procedure. The Coordinated Universal Time (UTC) time shifts between the Global Positioning System (GPS) and the two Picarro instruments were corrected for each instrument in addition to the inlet delay.

The instruments were installed on the back seat of a 2012 Volkswagen Transporter. One-quarter inch Teflon tubing was used to pull in air either from the front bumper (0.5 m above ground level) to the G2301 or from the rooftop (2 m above ground level) to the G4302. To avoid dust in the inlets for both instruments, an Acrodisc® syringe filter, 0.2 mm was used for the G2301 and Parker Balston 9933-05-DQ filter was used for the G4302. The G2301 was used for quantification and attribution purposes and the G4302 mainly for attribution. The vehicle locations were registered using a GPS system that recorded the precise driving track during each survey.

Katowice, Poland:

Picarro 2201-I or alternatively Los Gatos MGGA 918 was installed in the trunk of the passenger 4x4 car. Could be installed and de-installed within approx.3 min with quick swagelock connectors allowing to deliver the air from the roof of the car using 1/8 inch tube with a water droplet separator installed at the intake. Usual delay time between the methane concentration peak and recorded signal by the Picarro instrument was 35sec and is mostly affected by internal volume and flow rate of the pump installed in the analyser. In case of Los Gatos instrument the delay time was only 7seconds. The 7 inch diameter monitor is installed on the front passenger seat to observe the status of instrument and current reading from the analysers. Power system is instantaneous whenever batteries (Four Nerbo batteries NBC 100 – 12i 12V 100Ah) are loaded. Analysers were connected to MeanWell TN-1500 true sine wave inverter (which can deliver up to 1.5kW) The power was supplied by 2 or 4 batteries allowing for a 24h operation of Picarro without reloading. MeanWell PB-600-12 8 stage loading units allowing for the conditioning of the batteries). Los Gatos analyser was powered by 12V from the car installation only filtered from an unforeseen voltage peak.

London, United Kindgom:

Since May 2018, mobile surveys of London, UK have been conducted targeting each of the 32 boroughs of London, UK on a day-by-day basis, plus the boroughs of Runnymede and Spelthorne that are situated between RHUL and the boundary of Greater London, UK. All Uk surveys had a driving strategy of passing each km2 grid square of the British National Grid for a targeted location. Full coverage and completion

of the London, UK boroughs, plus the city center, were completed by October 2019. For early surveys, one km square with dense road network was selected for detailed coverage, but did not reveal more leaks as these tended to be focussed on the main road infrastructure.

The UK mobile surveys were conducted using a 4WD Dacia Duster SUV. A roof top mast is attached holding
3 inlet lines 1.8 m above the ground. One line is attached to a bag-sampling pump and the other two inlets are connected to two cavity ring-down spectrometer (CRDS) instruments located in the trunk of the vehicle. The mast also holds an anemometer and GPS unit that are connected to a Picarro G2301 analyzer which continuously measures [CH4]/[CO2]/ & [H2O]. This unit is coupled with an A0941 Mobile Module that matches the coordinates of the GPS unit located on the mast to the G2301 measurements. The second instrument is a Los Gatos Research Ultraportable Methane Ethane Analyzer (LGRUMEA) which measures concentrations of [CH4], [C2H6], & [H2O]. This measures at 0.5 Hz and has a standard flow rate of 1.7 L min-1. An internal Navilock-602U USB receiver records GPS coordinates which are later matched to the LGRUMEA measurements.

The inlet delay for the Picarro is about 9 seconds and the LGRUMEA is about 5 seconds. All instrument measurements and GPS coordinates are displayed either on the laptop or iPad through VNC connection with a mobile 4G network. On a monthly basis, both instruments are calibrated against NOAA (National Oceanic and Atmospheric Administration) cylinders on WMO reference scales.

Lowry et al., 2020 (https://doi.org/10.1016/j.scitotenv.2019.134600)
Zazzerie et al., 2015, 2017 (https://doi.org/10.1016/j.atmosenv.2015.03.029, https://www.nature.com/articles/s41598-017-04802-6)

Munich, Germany:

Measurements were taken with the same setup as in Hamburg, DE, however for Munich, DE we only used the Picarro G2301. Please see the descriptions for Hamburg, DE.

Paris, France:

Measurements were taken during from 17 surveys conducted between 07 September 2018 and 07 March 2019. The area surveyed includes Paris, FRwith its west and south suburbs (mostly Boulogne-Billancourt and Issy-les-Moulineaux), which have been extensively covered. Mobile measurements were provided by installing the analyzers in a vehicle equipped with a GPS device (NAVILOCK NL-602U). Walking measurements with portable instrument were made to obtain detailed information about the source of the observed enhancements. The AirCore tool (e.g. Defratyka et al., 2021), which was a part of the car mounted instrument, was used to determine the isotopic composition. Measurements during this study were made using cavity ring-down spectrometers (CRDS) manufactured by Picarro (Santa Clara, California) and a Los Gatos Research (LGR) analyzer (San Jose, California) model MGGA for more walking surveys. CRDS G2201-i was used almost all the time (except 06.03.2019 when two G2401 were used). All measurements are time-corrected to account for the delay induced by the travel time from the inlet (synflex 1/4") to the analyzers. Delays vary between 20 s and 30 s. Vehicle-mounted instruments had air inlets situated on the roof of the car. The potential influence of the inlet position of the observed CH4 mole fraction was tested. During three days, two similar instruments used different inlets. One inlet was installed on the roof of the car (~170 cm above the ground) and the second on the upper skirt of the car

(~50 cm above the ground). No significant difference was observed (Supement Information in Defratyka et al., 2021).

Between September 2018 and March 2019, a 3-point concentration and isotopic composition calibration was done for CRDS G2201-i. Calibration factors are hereafter applied. Additionally, a known gas was measured for 20 minutes before and after 11 randomly-selected surveys to check the analyzer stability and the influence of switching on/off the analyzer on the CH4 and δ13CH4 values. In all cases, the analyzer was stable and no detectable influence of switching on/off instrument was observed. The LGR MGGA analyzer was also tested and calibration factors applied.

Not included in the publication: 5 days of measurements with CRDS G2201-i, focused on the previously covered area. The same set-up as previously.

Swansea, United Kingdom:

The urban areas of Swansea, UK were surveyed in early March 2019. This consisted of 2 days of surveys. For mobile measurement setup see descriptions for London, UK.

Toronto, Canada:

From August 2018 to July 2019, mole fractions of CH4, CO2 and H2O are simultaneously measured on the car-based platform by a G1301 analyzer developed by Picarro (Santa Clara, California, USA). The G1301 analyzer is based on cavity ring-down spectroscopy (CRDS) and its stated precision is 1.5 ppb, 0.2 ppm over a 30 s integration period for CH4, CO2, respectively. From September 2019 to March 2020, mole fractions of CH4, CO2, CO and H2O are measured simultaneously on the car-based platform by a G2401 analyzer developed by Picarro. Its stated precision is 1 ppb, 50 ppb, 15 ppb over a 5 s integration period for CH4, CO2, CO respectively. Both CH4 and CO2 mole fractions collected with this analyzer are calibrated against the same two tanks. The sampling inlet was mounted on a mast 2.5 m above the ground.

Mole fractions of CH4, CO2 and H2O are measured simultaneously on the bicycle-based platform by an ultraportable multi-gas carbon emissions analyzer developed by Los Gatos Research (Mountain View, California, USA). This analyzer is based on Off-Axis integrated cavity output spectroscopy (QA-ICOS) and its stated precision is 3 ppb, 0.4 ppm over a 1 s integration period for CH4, CO2, respectively. The bicycle platform is coupled with an Airmar 220WX weather station.

The temporal offsets between the moment when the air is sampled by the inlet and when it reaches the cavity of the analyzer are carefully calculated by injecting air with enhanced mole fraction into the inlet and timing the response of the instrument.

We use two different sensors for the wind measurements. From August 2018 to the end of October 2018, we used an AIO 2 sonic weather sensor (Met One Instruments, Grants Pass, Oregon, USA) and a 220WX weather station after November 2018. A correction is applied to the wind data collected with the AIO 2 for both direction and speed to remove the impact of the vehicle's motion. The stated accuracies for the wind speed and direction for both sensors are 0.5 m/s and 5°, respectively. The platform coordinates were recorded using the GPS in the 220WX weather station when available or the GPS in a Garmin watch. The stated accuracy of each GPS receiver is 3 m but larger errors have been observed in the downtown area where the roads are surrounded by tall buildings, limiting GPS reception.

Utrecht, The Netherlands:

Please see the descriptions for Hamburg, DE.

S2. Definition of urban domains for road length and population calculation

	LLC (lat_deg)	LLC (lon_deg)	ULC (lat_deg)	ULC (lon_deg)	LRC (lat_deg)	LRC (lon_deg)	URC (lat_deg)	URC (lon_deg)
Barcelona, ES	41.3	2.08	41.46	2.08	41.3	2.23	41.46	2.23
Birmingham, UK	52.46	-1.92	52.52	-1.92	52.46	-1.72	52.52	-1.72
Bucharest, RO	44.38	26	44.48	26	44.38	26.18	44.48	26.18
Groningen, NL	53.18	6.48	53.25	6.48	53.18	6.62	53.25	6.62
Hamburg, DE	53.45	9.8	53.7	9.8	53.45	10.2	53.7	10.2
Katowice, PL	50.2	18.75	50.4	18.75	50.2	19.25	50.4	19.25
London, UK	51.3	-0.5	51.65	-0.5	51.3	0.25	51.65	0.25
Munich, DE	48.09	11.45	48.21	11.45	48.09	11.65	48.21	11.65
Paris, FR	48.8	2.2	48.92	2.2	48.8	2.43	48.92	2.43
Swansea, UK	51.6	-4.05	51.68	-4.05	51.6	-3.85	51.68	-3.85
Toronto, CA	43.59	-79.55	43.82	-79.55	43.59	-79.16	43.82	-79.16
Utrecht, NL	52.05	5.07	52.12	5.07	52.05	5.16	52.12	5.16

Table S1: Lower left corner (LLC), lower right corner (LRC), upper right corner (URC) and upper right corner (UPC) used to define the rectangular area analyzed for each city.

S3. Review of other urban methane surveys

To our knowledge, the first study published of an urban methane survey using high precision laser based analyzer was by Phillips et al. in 2013, documenting their survey efforts in Boston, MA, US (28). They utilized a Picarro G2301 analyzer with an additional preliminary pump to increase tubing flow throughput from their front bumper mounted (0.3m) inlet. They defined a leak to be any observed Methane mole fractions above 2.5ppm, and they binned together observations within 5m. They also reported statistics for observed above 5ppm, and 10ppm. This same experimental setup and analysis technique was used by subsequent survey studies in Washington, DC (25): and utilizing a Picarro 2132i instrument in Durham, NC, US; Cincinnati, OH, US; and Manhattan, NY, US (26). This study by Gallagher et al. also introduced a fourth category for observed leaks, those above 25ppm, and compared the observations from the previous studies. We refer to this grouping of leak definitions as the Phillips 2013 method of leak classification. A survey of Ithaca, NY was conducted by Chamberlain et al. which used Picarro G2201 with a front bumper and auxiliary pump. In this study, leaks were defined to be measurements in excess of 1.93ppm, which was the 98th percentile of observed measurements (33). None of their observed concentrations exceeded 2.32ppm CH_4, precluding them from reusing previous leak definitions.

Mobile methane mapping studies using a Picarro 2301 sampling a roof mounted inlet were completed in Indianapolis, IA, US and were briefly discussed (21), but these data were largely focused on measuring point sources, and therefore leak rate metrics were not determined. Similar methane surveys were also conducted in Beijing, China using Picarro G1301 and 2401 analyzers measuring a roof mounted inlet. This study focused on classifying CO_2:CH_4 ratios of known point sources, and also did not calculate leak rate metrics (33).

Hopkins et al. used a mobile laboratory equipped with a variety of instruments, including a Picarro G1301 to measure air from a mast mounted inlet 3.5m above ground in Los Angeles, CA, US (24). In their analysis, all data within 5s, and across 150 long road segments were binned together, and methane hotspots were defined as road segments with concentrations above the 95th percentile which were not associated with known point sources. They completed an analysis of observed tracer ratios to infer source attribution the hotspots, but individual leaks were not explicitly considered or quantified.

Von Fischer et al. and Weller et al. both analyzed observations from campaigns where Google Street View cars were equipped with Picarro 2301 analyzers measuring front bumper mounted inlets (6,7). Both studies employ empirical algorithms to quantify emissions from observed concentration enhancements above background measurements. The second version of the algorithm defined in Weller et al. is the method we use to compare observations in this work, log-log equation which only considers the observed concentration peak above background (7). For both studies small, medium, and large leaks were defined in as twice observed methane emissions within 20m spacing, with inferred emissions of <6Lmin-1, between 6Lmin-1 and 40Lmin-1, and >40Lmin-1, respectively. Results from surveys in Burlington, VT; Indianapolis, IN; Boston, MA; Staten Island, NY; and Syracuse, NY are presented in von Fischer et al. (6). Further survey results published in Weller et al. include surveys in Birmingham, AL; Pittsburgh, PA; and Dallas, TX (7). We refer to these leak definitions as the von Fischer 2017, and Weller 2019 methods of leak classification.

Urban methane leak surveys were also published for three Connecticut, US cities, Hartford, Danbury, and New London, UK in a study which utilized a Picarro G2301 measuring air from a filtered inlet placed outside the passenger side of the vehicle (30). In this study, leaks were defined statistically using a modified Tau approach. Confirmed outlier methane observations within 30m were binned together. We refer to this method of leak classification as the Keyes 2020 method.

S4. Comparing Inlet Height Differences and Weller 2019 Method Uncertainties

For five days in 2018 and 2019, the Toronto, CA group completed a methane mapping surveys with two gas analyzers, the LGR UGGA and Picarro G1301, with inlets mounted in different positions. On 2018-11-28, both instruments sampled from the vehicle mast at a height of approximately 2.5m. On this survey, both instruments recorded 41 coincidental enhancements, defined as enhancements recorded from each instrument within 20 seconds of the other. 17 of these enhancements were recorded to have a plume width within 200 m. Fitting a linear regression between these filtered coincidental enhancements demonstrates that the Picarro G1301 measured enhanced concentrations 1.3 times greater than the UGGA, which we argue is primarily due to the difference in instrument response.

For the rest of the coincidental survey days, conducted in April 2019, the mobile laboratory was reconfigured with the Picarro G1301 measuring air from an inlet from the front bumper of the vehicle, approximately 0.3m, while the UGGA sampled from the 2.5m mast inlet. During these surveys, 40 coincidental plumes with recorded widths within 200m. Fitting linear and proportional regressions to these plumes shows that measured concentrations with the G1301 were 1.4 times greater than the UGGA. This suggests that of the 40% increase in observed concentrations by the G1301, about 30% can be explained by the UGGA's slower instrument response, and the residual 10% can be attributed to the inlet height difference.

The main equation of the Weller 2019 algorithm can be written as a simple exponential function,

$$Q = e^{\left(\frac{0.988}{0.817}\right)} \cdot x^{\frac{1}{0.817}},$$

where Q is the methane emission rate in (L/min), and x is the maximum methane enhancement above the background. Therefore, a scaling error of y% in the input x, results in a y1/0.817% increase in determined emissions rates, Q. However, an offset error of ±Δx in the input x would change the emission rate by (Δx/x)1/0.817 %.

Therefore, a 10% decrease in concentration enhancements as measured by a 2.5m mast-mounted inlet when compared to a 0.3m bumper level inlet, would result in approximately 12% reduction in total emissions, as calculated by the Weller et al. 2019 algorithm.

References provided in the main text, additional reference for supplemental only:

33. Sun, W., Deng, L., Wu, G., Han, P., Miao, Y., Yao, B. Atmospheric Monitoring of Methane in Beijing using a Mobile Observatory. Atmosphere 2019, 10 (9), 554.
https://doi.org/10.3390/atmos10090554

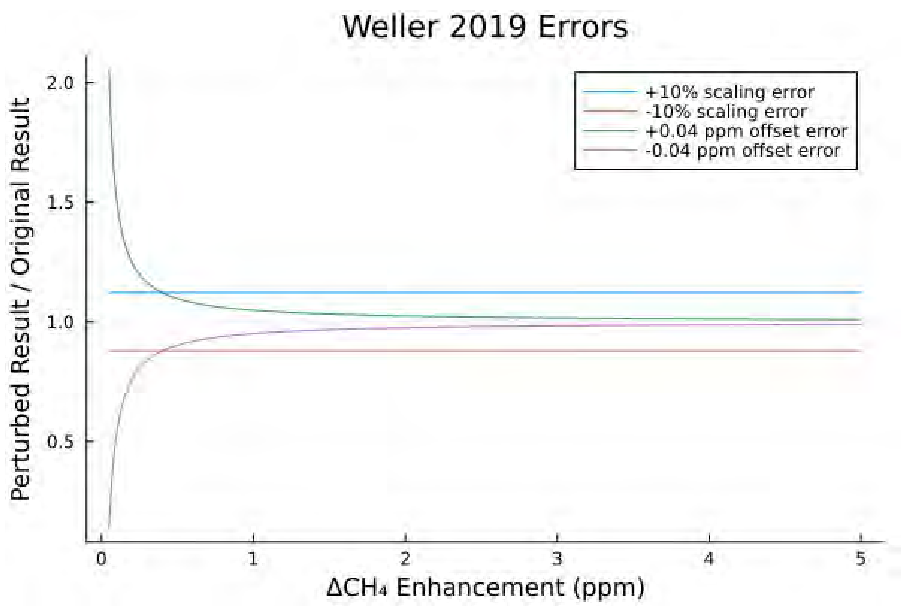

Figure S1. Biases caused by relative and absolute errors on emission rate derived from maximum local enhancement following Weller et al. 2019 method.

S5 Table for inventory-based estimates of city-wide emissions for methane from oil and gas distribution.

		National scale					City scale		
**City**	**ISO3**	**Marcogaz 2018 estimate CH4 emission (ton)**	**Country CH4 emissions NatGas distribution 1B2b5, 2018 (ton)**	**Total country population**	**Country population in urban area**	**Share of country population in urban area (%)**	**CH4 emissions urban area (ton) - CRF**	**CH4 emissions urban area (ton) - MarcoGaz**	MG/CRF
Hamburg	DEU	92,175	86,965	80,854,418	1,286,862	1.6%	1,384	1,467	106%
Utrecht	NLD	21,987	5,556	16,923,311	246,766	1.5%	81	321	396%
Paris	FRA	33,069	23,341	64,444,919	2,989,176	4.6%	1,083	1,534	142%
Bucharest	ROU	3,097	12,974	21,666,350	1,230,817	5.7%	737	176	24%
London	GBR	57,374	136,243	64,088,222	5,298,179	8.3%	11,263	4,743	42%
Groningen	NLD	21,987	5,556	16,923,311	185,367	1.1%	61	241	396%
Katowice	POL	28,038	22,969	38,562,189	1,258,714	3.3%	750	915	122%
Swansea	GBR	57,374	136,243	64,088,222	171,408	0.3%	364	153	42%
Birmingham	GBR	57,374	136,243	64,088,222	320,416	0.5%	681	287	42%

Table S2. Please see section 2.5. for further details on the rationale for the calculations.

S6 From Marcogaz report, 2018. The Technical Association of the European Gas Industry, Survey methane emissions for gas distribution in Europe.

Country EU28	Distribution network length total (in km)	PE (km)	Steel (km)	Cast Iron (km)	PVC (km)	Other (km)	ref.	Connection points (#)	ref	City Gates (#)	ref
**Austria**	43.400	28.683	11.423	2	1.814	1.478	1	1.349.000	3		2
**Belgium**	71.609	52.561	16.971	430		1.647	1	3.156.000	1	190	2
**Czech Republic**	73.181	42.445	30.736	0	0	0	1	2.849.000	3	4.254	2
**Denmark**	18.229	15.677	2.552	0	0	0	1	421.117	2	502	2
**Germany**	498.500	254.235	218.343	11.964	13.958	0	1	20.979.000	3	49.119	report
**Ireland**	11.339	11.226	113	0	0	0	1	661.000	3	89	2
**Italy**	257.844	72.067	181.690	2.897		1.190	1	23.203.000	3	7.000	2
**the Netherlands**	125.000	21.250	18.750	3.750	78.750	2.500	1	7.152.000	3	1.009	2
**Poland**	170.900	68.360	102.540	0	0	0	1	6.852.000	3	751	2
**Portugal**	17.450	15.339	2.094	17	0	0	1	1.382.000	3	73	2
**Slovakia**	33.301	14.519	18.782	0	0	0	1	1.506.000	3	1.760	2
**Spain**	70.307	59.761	9.281	1.266	0	0	1	7.556.000	3		2
**France**	203.092	143.003	53.157	5.681		1.251	2	11.268.000	3	3.731	2
**Finland**	1.911	1.808	83	20		0	2	31.000	3	0	2
**Slovenia(1)**	4.342	2.229	1.700	108	235	70	2	136.000	3	68	2
**UK**	126.335	81.657	7.242	17.362		20.074	2	23.184.315	2	49	2
**Greece**	6.087	4.663	1.281	136		0	2	325.000	3	24	2
**Romania**	17.218	8.958	8.260	0		0	2	1.408.170	2		
**Cyprus**	0	0	0	0	0	0		0	3		
**Latvia(1)**	5.500	2.824	2.153	137	298	89	4	443.000	3		
**Estonia (1)**	2.150	1.104	842	54	116	35	5	52.000	3		
**Lithuania(1)**	8.300	4.261	3.249	207	449	134	6	562.000	3		
**Croatia(1)**	18.386	9.439	7.197	458	996	296	7	647.000	3		
**Malta(1)**	0	0	0	0	0	0		0	3		
**Sweden(1)**	2.720	1.396	1.065	68	147	44	8	37.000	3		
**Bulgaria(1)**	249	128	97	6	13	4	9	74.000	3		
**Luxembourg(1)**	1.962	1.007	768	49	106	32	10	86.000	3		
**Hungary(1)**	84.000	43.124	32.879	2.094	4.548	1.354	11	0	3		
**Total**	**1.873.312**	**961.723**	**733.246**	**46.706**	**101.431**	**30.198**		**115.319.602**		**68.619**	

(1) For all pipeline lengths were no material lengths were available the average distribution of the known data was assumed.

Table 8: Activity factors for EU28 DSOs

Page 19 of 31

© MARCOGAZ WG-ME-17-25

Table S3 : Marcogaz data for European natural gas distribution grid
